# Experiential learning for children’s dental anxiety: a cluster randomized trial

**DOI:** 10.1186/s12903-020-01204-5

**Published:** 2020-07-31

**Authors:** Minmin Zhu, Hongbing Yu, Bo Xie, Hongwen Li, Qian He, Huimin Li, Jing Su, Xueqi Li

**Affiliations:** 1 Shenzhen Nanshan Center for Chronic Disease Control, Shenzhen, 518054 China; 2 Shenzhen Nanshan Maternal and Child Health Care Hospital, Shenzhen, 518054 China

**Keywords:** Dental anxiety, Child dentistry, Experiential learning, Efficacy, Tell-show-do, Cluster randomized trial

## Abstract

**Background:**

Dental anxiety (DA) has an impact on the quality of dental treatment and may have long-lasting implications for children. A recent study introducing experiential learning (EL) into children’s oral health promotion resulted in better oral hygiene. The purpose of the study is to evaluate whether EL can reduce children’s DA.

**Methods:**

In September 2018, we recruited 988 children aged 7–8 years from 24 classes to participate in a cluster-randomized trial. Classes were randomly assigned to EL (in which children received a lively presentation on oral health and participated in a role play in a simulated dental clinic in the classroom) or the Tell-Show-Do (TSD) group (in which children received a conventional TSD behavior management). The primary outcome was the prevalence of high DA after the procedure of pit and fissure sealant (PFS), assessed by a modified Children’s Fear Survey Schedule-Dental Subscale. Secondary outcomes were changes in blood pressures (BP) and pulse rates (PR) before and after the PFS procedure. The intervention effects were estimated by means of mixed effect models, which included covariates of gender and school (and baseline value for BP and PR only), and a random cluster effect.

**Results:**

In 396 children of the EL group who received the PFS treatment, the prevalence of high DA (score ≥ 38) was 18.5%, compared with 24.3% in 391 children of the TSD group (OR = 0.65; 95% confidence interval, 0.46–0.93; *P* = 0.019). The increases in BP and PR after the PFS were also significantly less in the EL group.

**Conclusion:**

School-based experiential learning intervention before a dental visit is feasible and effective in reducing children’s dental anxiety during PFS.

**Trial registration:**

The trial was registered in Chinese Clinical Trial Registry on 5 January 2020 (No.: ChiCTR2000028878, retrospectively registered).

## Background

Dental anxiety (DA), abnormal fear or dread of visiting the dentist and unwarranted anxiety over dental procedures, has an impact on the quality of dental treatment and may have long-lasting implications for children [1]. Cross-sectional and cohort studies published from 2000 to 2014 have reported prevalence of children’s DA that ranged from 10 to 20% [2]. DA sometimes leads to a series of uncooperative or disruptive behaviors before or during dental procedures, termed dental behavior management problems (DBMP), that result in stressful and unpleasant experiences for both the child and the dentist [3]. In addition, studies have shown that DA has a consistent impact on pain throughout the entire dental treatment [4], and DA/DBMP are associated with children’s dental caries [5–8], resulting in a vicious cycle [9].

DA varies from very mild to extreme levels, and interacts with urgency of treatment, therefore different approaches to anxiety reduction may be appropriate given the level of anxiety [10, 11]. Although it is possible to employ pharmacological interventions to manage high level of anxiety, such as anesthesia or sedation [12], dentists generally use communicational, behavioral and psychological techniques to manage children’s low or moderate level of DA and achieve a high quality of dental care. These include methods such as “Tell-Show-Do” (TSD), voice control, distraction, modelling, restraint, and cognitive restructuring [13, 14]. Of these methods, however, some need specialist training, some cause psychological traumas to children, and most are only initiated just before or during dental treatment. In recent years, the most used non-pharmacological method has been TSD, which has generally been acceptable for the doctors, children and parents [15–18]. Nevertheless, the TSD is less effective than modelling [19, 20].

Experiential learning (EL) is an innovative learning technique by which knowledge or skill is gained through the experience of participating in real or simulated practical activities [21]. EL is based on the theory that one can generalize an experience into a conceptualization through reflective observation and then proceed to take action. Active and personalized learning are characters of EL. EL has been used effectively in enhancing knowledge, improving attitude and modifying behaviors in health education [22–24], and was recently introduced into children’s oral health promotion, resulting in better oral hygiene [25].

Lack of control and unpredictability of the dental experience may be the dominant cause of DA [11]. By letting children participate in simulated dental activities before real treatment, EL is expected to help children develop knowledge of dental health and be familiar with the dental procedures, thus reduce DA among children. In this paper, we developed a school-based EL intervention and evaluated whether the EL was effective in reducing DA in primary school children.

## Methods

### Study design

This was a 2-arm cluster-randomized control trial comparing EL and TSD for their effectiveness in reducing DA in children. This study had the approved by the Ethics Committee of the Shenzhen Nanshan Maternal and Child Health Care Hospital. All parents of the children participated provided written informed consent. The trial was registered in Chinese Clinical Trial Registry (ChiCTR2000028878).

### Participants

The participants were children aged 7–8 years, selected in September 2018 from the second grade of primary schools in the Nanshan District of the city of Shenzhen, China. At this age, children in Shenzhen routinely receive pit and fissure sealant (PFS) as a prophylaxis for dental caries in permanent molar teeth, which is noninvasive procedure. Although PFS is harmless, children still show anxiety when facing the fissure sealant and its metal tip [26].

### Recruitment

**Fig. 1:**
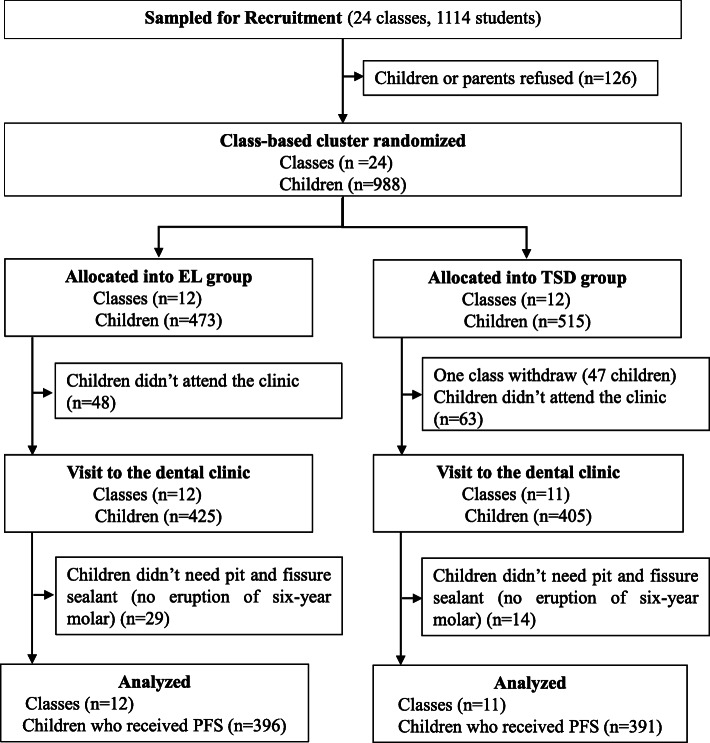
Flow diagram of the trial of EL vs TSD for reducing children’s dental anxiety. EL: Experiential learning, TSD: Tell-Show-Do

The eligibility criteria for second grade classes were: 1) located in Nanshan District, Shenzhen City; 2) had 40 to 50 children aged 7–8 years; 3) agreed to participate. In September 2018, we used a multi-stage random sampling method to recruit the participants. We first used a table of random digits to select six primary schools in Nanshan that had four or more classes met eligibility criteria, and then random selected four eligible classes in each of the six schools. All children in the 24 selected classes were invited to take part in the study, and the informed consent were sent to their parents (Fig. 1). Children who already had a dental visit or refused to participate were excluded.

### Randomization and interventions

In this trial, the class was the unit of randomization. The sampled classes were randomly assigned into the EL or TSD groups in 1:1 ratio in school blocks with the use of a table of random digits (Fig. 1). Cluster randomization was necessary to avoid between-group contamination and it was feasibly conduct school-based intervention. After receiving the signed informed consent from the parents, the intervention was implemented on a class by class basis. Neither the dentists, nurses nor field interviewers who assessed the outcomes were aware of the randomization assignments of the classes, as the measurements were the same for both groups. Only the dental nurses who performed the “Tell and Show” procedures were aware the assignments.

At the beginning of intervention, children in the EL group received a lively 40-min presentation on oral health given by dentists. The standardized oral health education conveyed information about dental caries and their causes, proper toothbrushing and flossing, and the pit and fissure sealant, by using slides, cartoon videos, and dental models. One month later, the children participated in an EL activity in a simulated dental clinic in the classroom. In the activity, the dentists demonstrated the common tools of dentistry (mouth mirror, dental probe, dental handpiece and dental ejector, etc.). Then the children were organized in 4-person-groups for a five-minute role play around a dental bed, with which the children playing a patient, a dentist, a nurse and a parent. While one group of children performed their role play, the other children were in another room watching cartoon videos that delivered oral health education. And another month after that, they visited the dental clinic to receive the PFS treatment.

Children in the TSD group received a conventional 5-min TSD behavior management session at the time of their visit to the dental clinic for the PFS procedure. The TSD behavior management process used in this study contained three procedures: Tell, a dental nurse explained to the child what the dentist would do during the PFS treatment; Show, the dental nurse showed the equipment involved; Do, the dentist performed the PFS procedure.

### Trial measurement

Another dental nurse (not the same one who delivered the TSD) performed the measurements of the children’s blood pressures and pulse rates with an upper-arm electronic sphygmomanometer in the waiting room after a 15-min quiet sitting period before the PFS treatment, and then remeasured them again after the treatment.

The field interviewers administered the modified Children’s Fear Survey Schedule-Dental Subscale (Modified CFSS-DS) [27] to the children after the PFS. The Modified CFSS-DS was translated into Chinese from the original English version and was combined with a facial image scale (FIS) [28]. It had a Cronbach’s alpha of 0.85 and a test-retest reliability intraclass correlation coefficient of 0.73 [27].

### Outcomes

The primary outcome was the prevalence of high DA, defined as a sum score of Modified CFSS-DS equal or above 38 [2]. The secondary outcomes were mean differences of change in systolic blood pressures (SBP), diastolic blood pressures (DBP) and pulse rates (PR) in the measurements taken before and after PFS.

### Statistical analysis

With an enrollment of 12 class per trial group, 40 participants per class, a rate of cohort retention of 80%, a rate of PFS of 90%, and an estimated intraclass correlation coefficient (0–0.01), the initial design yielded 80% power to detect a 10% difference in the prevalence of high DA between TSD and EL groups, with a two-sided alpha level of 0.05.

The intervention effects were estimated by means of mixed effect logistic regression for prevalence of high DA and linear mixed-effects model for continuous variations (DA score, SBP, DBP and PR), which included a random cluster effect of class. The primary predictor was group (EL versus TSD) and included covariates of gender and school (and baseline value for SBP, DBP and PR only), which were correlated with the dependent variable. All analyses were performed with the R project (Version 3.6.1 Patched for × 64 Window system), and a *P* value < 0.05 was considered statistically significant.

## Results

### Characters of participants

A total of 24 classes from 6 schools were enrolled in the trial between Sept. 1st 2018 and Sept. 30th 2018, with a total initial enrollment of 988 children. The primary statistical analysis was based on the 396 participants in 12 EL classes and 391 participants in 11 TSD classes who completed the EL or TSD and the PFS treatment (Fig. 1). The two groups were similar in gender distribution and register residence (Table 1).

**Table 1:** Character of trial participants completing EL or TSD and PFS

Characters	EL group (***n*** = 396)	TSD group (***n*** = 391)	***P*** value^**$**^
Gender			0.623
Male	221	226	
Female	175	165	
Register Residence^a^			0. 654
Shenzhen	255	259	
Others	124	116	

*EL* Experiential learning, *TSD* Tell-Show-Do, *PFS* Pit and fissure sealant;

^a^The register residence was unknown for 17 and 16 children in the EL and TSD groups, respectively

^$^*P* value was calculated with the chi-square test

### Primary outcome

The mean score of DA was 27.3 (Standard Deviation [SD]: 11.6) in the EL group and 29.2 (SD: 12.1) in the TSD group, with an adjusted mean difference of − 2.5 (95% confidence interval [CI]: − 4.9 − − 0.2, *P* = 0.042). The prevalence of high DA (Modified CFSS-DS scores ≥38) was 18.5% in the EL group and 24.3% in the TSD group. The mixed effect logistic regression showed that the OR was 0.65 (95% CI: 0.46–0.93; *P* = 0.019), after adjusting gender and school (Table 2).

**Table 2:** Primary and secondary outcomes

Outcomes	EL group	TSD group	Intervention effect^**a**^	***P*** value^**$**^
**DA (score)**	27.3 ± 11.6	29.2 ± 12.1	−2.5(− 4.9 − − 0.2)	0.042
**High DA (%)**	18.5	24.3	0.65(0.46–0.93)	0.019
**SBP (mmHg)**				
At the waiting room	108.7 ± 12.1	107.8 ± 12.7		
After the PFS	106.1 ± 13.0	108.5 ± 14.2		
Change	−2.7 ± 12.8	0.7 ± 14.2	−2.6(−4.4 − −0.9)	0.036
**DBP (mmHg)**				
At the waiting room	66.2 ± 9.5	65.5 ± 10.0		
After the PFS	68.0 ± 12.1	70.0 ± 11.6		
Change	1.7 ± 12.3	4.5 ± 12.9	−2.0(−4.4–0.3)	0.086
**PR (Beats per minute)**				
At the waiting room	93.1 ± 14.0	92.0 ± 13.5		
After the PFS	93.3 ± 13.9	94.4 ± 13.4		
Change	0.2 ± 14.0	2.5 ± 12.2	−1.7 (−3.4 − −0.1)	0.038

*DA* Dental anxiety, *SBP* Systolic blood pressures, *DBP* Diastolic blood pressures, *PR* Pulse rates, *EL* Experiential learning, *TSD* Tell-Show-Do, *PFS* Pit and fissure sealant; Plus–minus value is Mean ± SD

^a^For prevalence of high DA, the result is the odds ratio and 95% confidence interval; for DA score, SBP, DBP and PR, the results are the mean difference and 95% confidence interval

^$^For prevalence of high DA, the *P* value was calculated with a mixed-effect logistic regression, which included a random cluster effect of class, and covariates of gender and school; while for difference of DA score, SBP, DBP and PR, *P* values were calculated by linear mixed-effect models, with a random cluster effect of class, and covariates of gender, school and baseline value (for SBP, DBP and PR only)

### Secondary outcomes

The mean SBP measured in the waiting room before the PFS was 108.7 mmHg in the EL group and 107.8 in the TSD group. The mean change of SBP from before to after the PFS was − 2.7 in the EL and 0.7 in the TSD group, with a − 2.6 mmHg adjusted mean difference (95% CI: − 4.4 to − 0.9 mmHg; *P* = 0.036). The mean DBP after the procedure increased in both groups, but by 1.7 mmHg in the EL group and 4.5 mmHg in the TSD group, an adjusted mean difference of − 2.0 mmHg (95% CI: − 4.4 to 0.3 mmHg; *P* = 0.086) (Table 2).

The mean PR in the EL group increased by 0.2 Beats Per Minute (BPM), while in the TSD group it increased by 2.5 BPM, for an adjusted mean difference of − 1.7 BPM (95% CI: − 3.4 to − 0.1 BPM; *P* = 0.038) (Table 2).

**Fig. 2:**
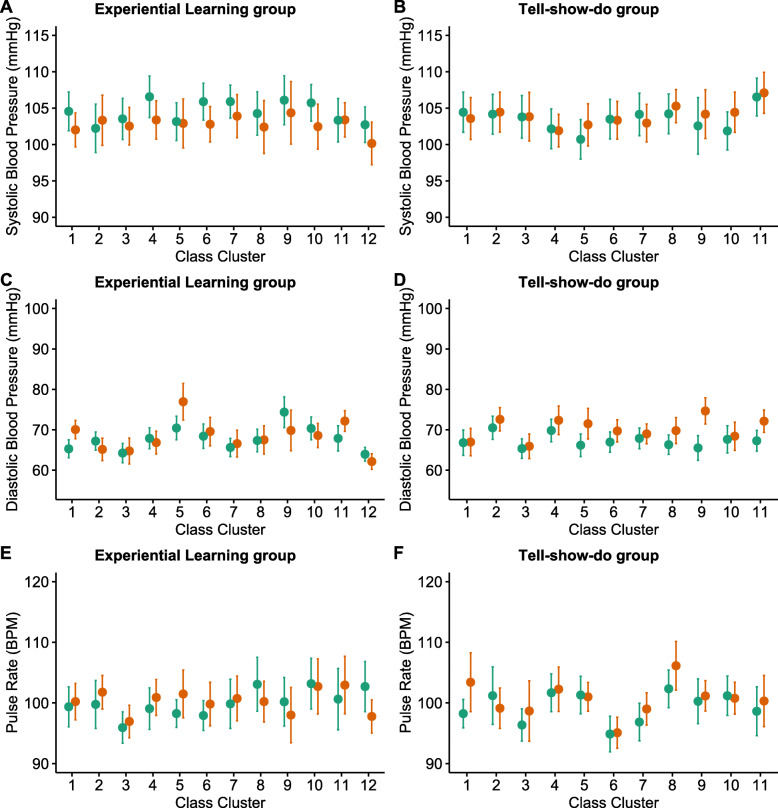
SBP, DBP and PR before and after PFS according to class clusters. SBP: Systolic blood pressures, DBP: Diastolic blood pressures, PR: Pulse rates, and PFS: Pit and fissure sealant. The dots indicate the mean values, and I bars indicate the upper confidence value (mean plus 1.96 times the standard error) and the lower confidence value (mean minus 1.96 times the standard error). Those with green color represent values before PFS, and red indicate values after PFS

Detailed data of SBP, DBP and PR before and after the PFS according to the class cluster were illustrated (Fig. 2).

## Discussion

To our knowledge, this is the first trial to assess the use of a school-based experiential learning intervention to reduce children’s dental anxiety. On average, there were fewer children with high dental anxiety, as defined by a score of ≥38 on the Modified CFSS-DS, in the EL group compared to the TSD group, with significantly lower increases in the SBP and PR after the PFS procedure.

There is evidence to suggest that classical conditioning plays a major role in the development of DA [1]. Children who have had a negative dental experience will have more DA [29], and can lead to DA in adulthood [30]. When enter an unfamiliar circumstance, the level of children’s anxiety will increase. In the conventional TSD management, the children only listened and observed the process of treatment immediately before they were subjected to and might not have been emotionally ready for the treatment.

To reduce high levels of dental anxiety over the long term, the most effective treatment is graded exposure [31]. In contrast to TSD, in this study, the EL intervention firstly educated the children about the dental procedures, then showed them the dental tools, and finally invited them to role play in a simulated dental clinic a month before their dental visit. Also, children would share their felling after the role play, a “reflective observation” on their experiences. It allowed them to become familiar with the common dental tools and experience a simulated dental procedure, but not to face unpredictable anxiety-inducing stimuli, making them gradually feel in control and will improve their self-efficacy. Similar to the EL intervention, Radhakrishna et al. modified the TSD technique by adding a component of learning through playing [32], and achieved lower DA scores.

In our trial we chose a cut-off value of 38 on the CFSS-DS to define high DA, which was also used by most studies in the systemic review by Cianettic et al. [2]. The reduction of high DA obtained in our study, although not achieving the designed 10 percentage-point difference, was similar to that obtained during moderately invasive treatment with other non-pharmacologic techniques such as using modelling [20] or audiovisual distraction [33].

The blood pressure and pulse rate, physiological indicators of anxiety during the dental procedure, would be expected to increase during the treatment and slowly descend after it concluded [34]. A study showed that TSD controlled increase of blood pressures in patients during dental therapy [35]. In this trial, EL intervention was more effective than TSD, as the SBP and PR increased less after the PFS in the EL group than in TSD group, and the mean differences of changes between the two groups were statistically significant.

Within various DA nonpharmacologic management techniques, some seek to enable dental treatment to be performed, and some seek to rehabilitate the dental anxiety. Physical restraint, distraction (diverts one’s attention away from auditory and/or visual unpleasant stimuli) [33], modeling (behavior guidance), and tell-show-do (reduces uncertainty) probably belong to the techniques of enabling treatment. While cognitive behavioral therapy (CBT), a combination of both cognitive restructuring and behavioral modification interventions, rehabilitates the dental anxiety. A systematic review reported that pediatric patients who received CBT, especial graded exposure, reduced their dental anxiety [31]. In this study, EL intervention helped the children be familiar with the dental procedures, enabling dental treatment to be performed. Meanwhile, through self-experience, reflection and graded exposure, EL restructured children’s negative cognition to dental therapy, which might help rehabilitate the dental anxiety.

Due to several considerations, we chose children in the second year of primary school as the target group for the EL intervention. These children have a high incidence of dental caries, some of them will probably visit the dentists soon. Also, they have better understanding than children in kindergarten. As it is right before the planned dental prophylaxis of pit and fissure sealing for the first permanent molar, the intervention can be conveniently executed and evaluated.

In contrast to the clinic-based intervention, school-based EL intervention can be conveniently performed at any time before the dental visit, on a large scale. School-based EL is effect in reduction of children’s DA during PFS, and different EL activities might need to be delivered in schools to cover other dental procedures than PFS. In Shenzhen, or other cities in China, the children receive health education presentations regularly at school. We believe that simply adding a special oral health education and short session of role playing in a simulated dental clinic in the class can have a significant effect in reducing DA in children.

The limitation of our study was that we adopted “Per Protocol analysis” but not the gold standard analysis method of “Intention to Treat”, this was due to missing outcomes for those children who did not attend the clinic and who did not need PFS. Also, it is possible that the reductions in DA, BP and PR varied depending upon which role the children played in the EL (i.e. dentist, nurse, parent or patient), but we were not able to evaluate this as we did not record roles for every child.

## Conclusion

School-based experiential learning intervention before a dental visit is feasible and effective in reducing children’s dental anxiety during the PFS. Not only will this make dental procedures easier and less traumatic for the dentists, nurses and children, but it could also reduce the potential for DA in adults.

## Data Availability

The datasets used and/or analyzed during the current study are available from the corresponding author on reasonable request.

## Abbreviations

BPM: Beats per minute
BP: Blood pressures
CBT: Cognitive behavioral therapy
DA: Dental anxiety
DBMP: Dental behavior management problems
DBP: Diastolic blood pressures
EL: Experiential learning
Modified CFSS-DS: Modified Children’s Fear Survey Schedule-Dental Subscale
PFS: Pit and fissure sealant
PR: Pulse rates
SBP: Systolic blood pressures
SD: Standard deviation
TSD: Tell-Show-Do
